# Oxidative Stress-induced Telomere Length Shortening of Circulating Leukocyte in Patients with Obstructive Sleep Apnea

**DOI:** 10.14336/AD.2016.0215

**Published:** 2016-10-01

**Authors:** Kyung Soo Kim, Jin Wook Kwak, Su Jin Lim, Yong Kyun Park, Hoon Shik Yang, Hyun Jik Kim

**Affiliations:** ^1^Department of Otorhinolaryngology, Seoul National University College of Medicine; ^2^Department of Otorhinolaryngology and Head & Neck Surgery, Chung-Ang University College of Medicine, Seoul, Korea

**Keywords:** obstructive sleep apnea, telomere length, oxidative stress, peripheral blood, leukocyte

## Abstract

The main mechanism of pathogenesis which causes systemic complications in obstructive sleep apnea (OSA) patients is believed to be intermittent hypoxia-induced intermediary effect and it depends on the burden of oxidative stress during sleep. We aimed to search the predictive markers which reflect the burden of systemic oxidative stress in patients with OSA and whether excessive telomere length shortening is a characteristic feature that can assess oxidative stress levels. We used quantitative PCR to measure telomere length using peripheral blood genomic DNA. Telomere lengths were compared in an age- and body mass index (BMI)-dependent manner in 34 healthy volunteers and 43 OSA subjects. We also performed reactive oxygen species assay to measure the concentration of hydrogen peroxide in the peripheral blood of healthy volunteers and OSA subjects. We found that the serum concentration of hydrogen peroxide was considerably higher in OSA patients, and that this was closely related with the severity of OSA. Significantly shortened telomere length was observed in the circulating leukocytes of the peripheral blood of OSA patients, and telomere length shortening was aggravated more acutely in an age- and BMI-dependent manner. An inverse correlation was observed between the concentration of hydrogen peroxide and the telomere length of OSA patients and excessive telomere length shortening was also linked to severity of OSA. The results provided evidence that telomere length shortening or excessive cellular aging might be distinctive in circulating leukocyte of OSA patients and may be an predictive biomarker for reflect the burden of oxidative stress in the peripheral blood of OSA patients.

Cellular aging is a natural process characterized by progressive functional impairment and decreased capacity to respond appropriately to environmental stimuli or injuries [1,2]. The ends of eukaryotic chromosomes have highly conserved hexanucleotide repeats (TTAGGG) known as telomeres. Telomeres are essential for genomic stability because they protect chromosome ends from DNA damage and prevent chromosomal end-to-end fusion. Somatic cells lose 50 to 150 bp of telomeric repeats with each round of DNA replication [3]. Excessive telomere shortening has been found in the circulating leukocytes of patients with age-related disorders, chronic inflammation, and hormonal or metabolic disturbance including obesity [1]. Although diverse molecular and cellular mechanisms have been proposed to be causes of excessive cellular aging, oxidative stress is believed to be one of the most potent factors of abnormal cellular aging through its influence on protein conformations, catalytic activity, protein-protein, and protein-DNA interactions. Therefore, it has been suggested that telomere length shortening in circulating leukocytes reflects the cumulative burden of systemic oxidative stress [2].

Obstructive sleep apnea syndrome (OSA) is characterized by repeated events of partial or complete airway obstruction that reduce or interrupt airflow and disrupt normal ventilation. In patients with untreated OSA, episodes of hypoxia occur frequently during each hour of sleep and can happen nightly for several decades, increasing the risk of systemic complications, such as hypertension, stroke, ischemic heart disease, arrhythmia, congestive heart failure, and atherosclerosis [4,5]. The increased risk of systemic complications in OSA patients is believed to be mediated by intermediary mechanisms such as sympathetic activation and oxidative stress due to the episodes of intermittent hypoxia [6-9]. Oxidative stress might lead to endothelial dysfunction, vascular inflammation, and insufficient blood supply to organs, which are major factors in the progression of systemic diseases [10,11]. Accordingly, more researches on biomarkers are needed to predictive the burden of systemic oxidative stress in OSA patients.

We hypothesize that intermittent hypoxia-induced oxidative stress in the peripheral blood of OSA patients promotes abnormal cellular aging in circulating leukocytes. Furthermore, the ability to detect oxidative stress or abnormal cellular aging in the peripheral blood can provide clinically useful information for predicting cardiovascular complications in OSA patients.

The purpose of the present study was to compare telomere length in the genomic DNA of circulating leukocytes from OSA patients and healthy volunteers and to assess the level of oxidative stress in the peripheral blood of OSA patients. We also sought to evaluate whether there is a correlation between telomere length shortening and the burden of oxidative stress in OSA patients.

## MATERIALS AND METHODS

### Study participants

This study enrolled 77 people who were referred to the Sleep Disorder Center in the Department of Otolaryngology and Head & Neck Surgery of Chung-Ang University College of Medicine (Seoul, Korea) between July 2012 and June 2014. Participation was voluntary, and written informed consent was obtained from all participants. The Institutional Review Board of Chung-Ang University College of Medicine approved this study (IRB number C2012248 [943]). The sleep reports and medical records of 34 healthy volunteers were retrospectively reviewed.

A polysomnography was used to diagnose OSA and assess OSA severity using the respiratory disturbance index (RDI) and oxygen desaturation index (ODI). Thirty-four healthy volunteers confirmed to have RDI and ODI within normal range were also evaluated. A total of 151 OSA patients were enrolled in the present study and 88 patients with smoking history, 68 patients with hypertension, 65 patients with allergic rhinitis, 52 patients with diabetes, 34 patients with asthma, patient who were on the medication for atherosclerosis (N=5), arrhythmia (N=7), and congestive heart failure (N=3) were excluded. Forty three OSA patients were included in the present study and their sleep reports and medical record were reviewed.

To assess the age-dependent influence on telomere length, subjects were classified by their age: who were younger than 20 years old, who were 20-40 years, who were 40-60 years, and who were older than 60 years. The subjects were also classified into their body mass index (BMI) to analyze weight-dependent changes of telomere length: who were less than 22.9 kg/cm^2^, who were 23.0 to 24.9 kg/cm^2^, and who were greater than 25.0 kg/cm^2^.

### Blood sample collection and measurement of telomere length

Two milliliters peripheral blood was collected from 77 participants. Genomic DNA was extracted from 2 ml whole blood using G-spin Genomic DNA Extraction Kits (iNtRON Biotechnology Inc, Seongnam-si, Kyeonggi-do, Korea). All DNA samples were diluted to the same concentration (based on ultraviolet absorbance) and stored at -80°C until use. Genomic DNA from peripheral blood was subjected to real time-PCR and the telomere length of chromosomes from whole blood DNA was measured as the telomere repeat copy number relative to the single gene copy number (T/S ratio). Real-time PCR was performed using a Light Cycler 2.0 (Roche Diagnostic, Mannheim, Germany), and the rate of accumulation of amplified DNA was measured by continuous monitoring with a LightCycler FastStart DNA Master SYBR Green (Roche Diagnostic, Mannheim, Germany) with MgCl_2_ at 2 mM.

Primers for PCR were as follows:

Telomere-F: 5’-GGTTTTTGAGGGTGAGGGTGAG GGTGAGGGTGAGGGT-3’.
Telomere-R: 5’-TCCCGACTATCCCTATCCCTATC CCTATCCCTATCCCTA-3’.
Beta-globulin-F: 5’-GCTTCTGACACAACTGTGTT CACTAGC-3’
Beta-globulin-R: 5’-CACCAACTTCATCCACGTT CACC-3’.

Telomere amplification occurred at 95°C for 10 min followed by 25 cycles of 95°C for 10 s and 58°C for 1 min. Beta-globin amplification occurred at 95°C for 10 min followed by 35 cycles of 95°C for 10 s and 56°C for 15 s. All samples were run in duplicate using 25 ng of DNA per 10 μl reaction. Quantitative values were obtained relative to the Ct value at which a single increase associated with exponential growth of PCR products was detected using the Light Cycler analysis software. Ct values were used to calculate the T/S ratio for each sample using the equation: T/S=2-ΔCt (where ΔCt = Ct of single-copy gene-Ct of telomere). Coefficients of variation (CVs) for telomeres, single genes, and T/S ratio duplicate assays were < 4%, < 3%, and < 5%, respectively. Mean values of telomere length were compared between healthy volunteers and OSA patients. Values from OSA patients were evaluated according to age, body mass index (BMI), OSA severity, and ODI dependence.

### Reactive oxygen species (ROS) concentration Assay

Two milliliters peripheral blood was collected from 77 participants and immediately aliquoted into cryotubes as plasma. Total ROS concentration in plasma from patients’ peripheral blood was subjected to ELISA using OxiSelectTM ROS assay kit (Cell Biolabs, San Diego, CA) to assess the mean concentration of hydrogen peroxide. The assay was performed as prescribed in the manufacturer’s instructions. Briefly, unknown ROS samples or standards were added to the wells with a catalyst that helps to accelerate the oxidation reaction. Samples were measured fluorometrically against a hydrogen peroxide or 2’ 7’-dichlorodihydrofluorescein (DCF) standard. The assay was performed in a 96-well fluorescence plate format that can be read on a standard fluorescence plate reader. The ROS concentration in unknown samples was determined by comparison with the predetermined DCF or hydrogen peroxide standard curve.

### Statistical analysis

Data are presented as the mean + standard deviation for normal distributions, median with interquartile range (IQR, 23-75th percentile) for non-normal distributions, or number (%) for categorical variables. Telomere lengths were logarithmically transformed prior to statistical analyses in order to approximate a normal distribution. Pearson’s correlation coefficients were calculated to evaluate the relationships between telomere length and continuous variables. Significance was defined at the 0.05 level of confidence. Stepwise multiple linear regression analysis was performed to exclude the influences of potential confounding variables. All analysis was performed with SPSS (version 18.0; SPSS Inc., Chicago, IL, USA) for Windows software.

**Table 1:** The concentration of hydrogen peroxide, Respiratory disturbance index (RDI), and mean telomere length of leukocytes in OSA patients (N=43)

OSA severity	Patient No	H_2_O_2_	RDI	Telomere length
Mild	1	2.86	13.3	1.091373
	2	1.81	8.1	3.584852
	3	2.03	6.5	2.821393
	4	2.84	8	2.512685
	5	1.71	5.1	2.213215
	6	2.83	12.7	1.227582
	7	1.81	6.8	3.15173
	8	1.55	8.1	3.743148
	9	1.92	10.4	2.477892
	10	1.04	7.4	2.808145
	11	1.44	6.9	2.714857
Moderate	1	2.83	25.7	1.442105
	2	2.59	22.9	0.78
	3	2.78	23.7	0.6534477
	4	2.77	24.4	1.325139
	5	3.12	21.8	2.307201
	6	1.82	16.5	1.766386
	7	1.73	16.8	1.844485
	8	2.96	19.6	0.9768059
	9	2.1	21.4	1.52453
	10	1.99	19.4	1.93459
	11	2.78	23.5	1.008529
	12	2.41	17.5	1.849059
	13	1.41	16.3	1.848659
	14	1.92	26.4	1.707028
	15	2.88	18.5	0.5581385
	16	2.01	19.2	1.931746
Severe	1	3.82	71.3	0.477892
	2	3.2	38.8	1.046915
	3	2.7	50.7	1.020168
	4	4.84	74.6	0.7610925
	5	2.53	36.3	0.6625695
	6	2.94	61.2	1.025369
	7	2.97	51.6	0.1077787
	8	3.74	68.7	1.17784
	9	3.13	58.3	1.041758
	10	3.41	49.7	0.781251
	11	2.98	39.5	0.442105
	12	3.76	52.5	0.65228
	13	3.1	60.6	0.98
	14	2.43	30.1	0.73
	15	2.39	47.2	1.93
	16	3.51	57.3	0.68

**Figure 1.**
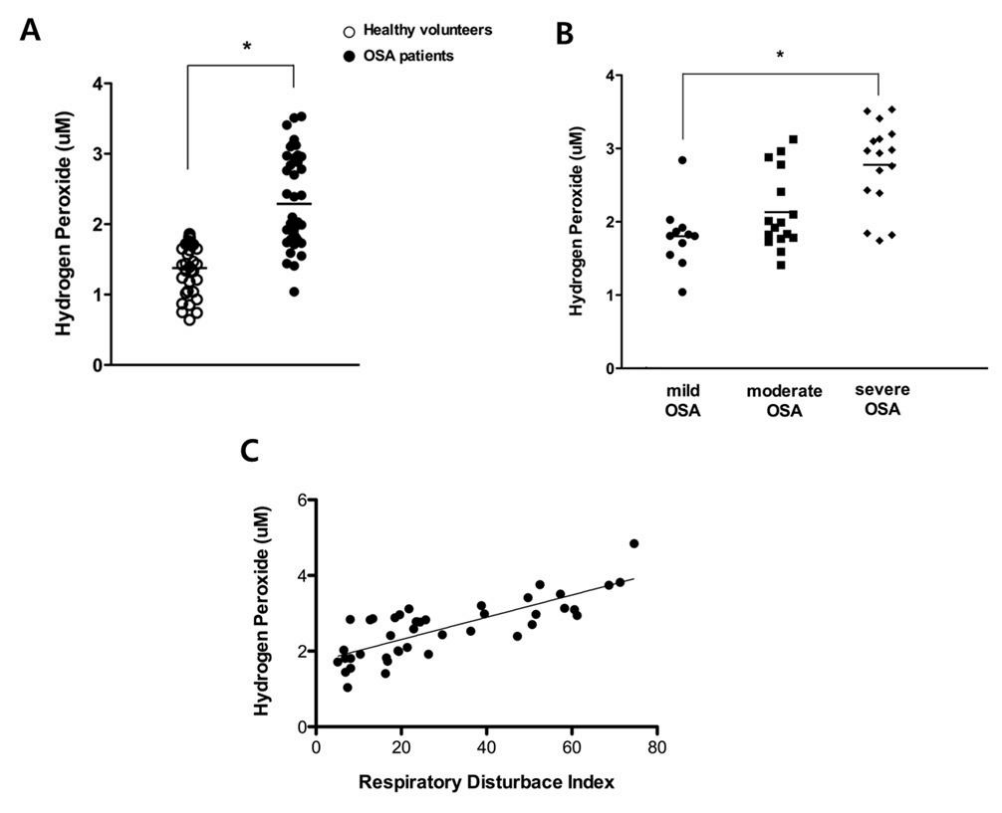
**The concentration of hydrogen peroxide in the peripheral blood of healthy volunteers (N = 34) and OSA patients (N = 43) and correlation with severity of OSA.** Mean concentration of hydrogen peroxide in the peripheral blood of healthy volunteers and OSA patients (A). Mean concentration of hydrogen peroxide in the peripheral blood of OSA patients compared by severity of OSA (11 mild OSA (circle), 16 moderate OSA (rectangle), and 16 severe OSA (diamond) patients) (B) and a significant correlation was observed between the concentration of hydrogen peroxide and the RDI of 43 OSA patients (R^2^=0.615) (C). Graphs show mean values. White dot: the concentration of hydrogen peroxide in healthy volunteers; black dot: the concentration of hydrogen peroxide in OSA patients (^*^p < .05 comparing healthy volunteers and OSA patients).

## RESULTS

### Characteristics of healthy subjects and OSA patients

Healthy volunteers included twenty-two men and 12 women with a mean BMI of 23.2 kg/m^2^ and mean age of 42.5 years. The OSA patients included 31 men and twelve women, with a mean BMI of 24.3 kg/m^2^ and mean age of 45.6 years. Mean values of age, sex, and BMI were not significantly different between healthy volunteers and OSA patients. Table 1 shows RDI, telomere length and the concentration of hydrogen peroxide in OSA patients. Healthy volunteers had RDIs of less than 5 and those with RDIs over 5 were included into the OSA patient group. OSA patients were classified into three groups by OSA severity. Among 43 OSA patients, eleven had mild OSA, sixteen had moderate OSA, and sixteen had severe OSA with mean RDIs for each group of 8.5, 20.9, and 53.0, respectively.

### The concentration of hydrogen peroxide was elevated in OSA patients’ peripheral blood

To examine the burden of systemic oxidative stress in OSA patients, we compared the mean concentration of hydrogen peroxide in patients’ peripheral blood with that of healthy volunteers. The results showed that the mean hydrogen peroxide concentration in the peripheral blood of healthy volunteers (N=34) was 1.38 + 0.4 uM and the hydrogen peroxide concentration was significantly higher in peripheral blood of OSA patients (N=43, 2.29 ± 0.7 uM, *p* < 0.05) (Fig. 1A). We assessed the correlation between the hydrogen peroxide concentration in OSA patients and OSA severity. The results showed that the hydrogen peroxide concentration in the peripheral blood increased with greater disease severity. Patients with severe OSA had the highest hydrogen peroxide concentration in their peripheral blood (2.78 + 0.6 uM) compared with mild OSA (1.80 + 0.4 uM, *p* < 0.05, Fig. 1B). In addition, the hydrogen peroxide concentration of the peripheral blood of OSA patients was proportionally related to their RDI (R^2^=0.615, p < 0.05 Fig. 1C). We found that OSA patients might be exposed to relatively higher oxidative stress in their peripheral blood in accordance with severity of disease and apneic events.

**Figure 2.**
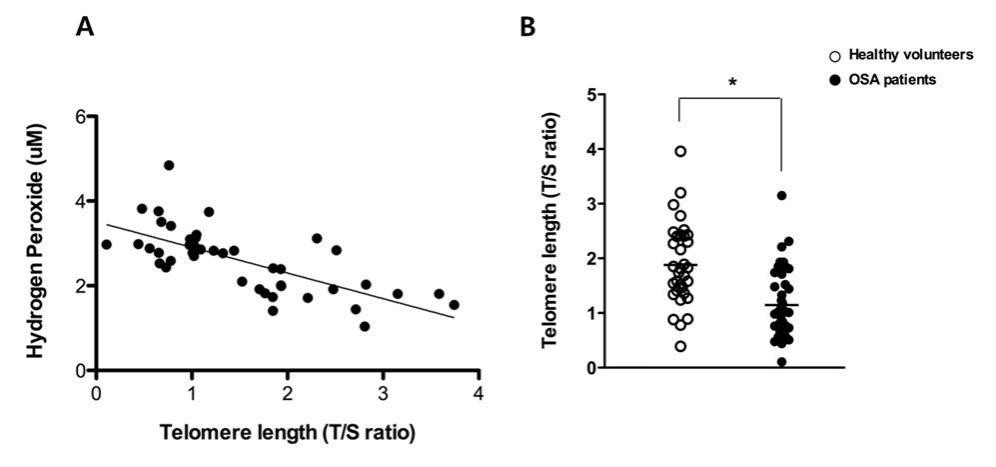
**Correlation between the concentration of hydrogen peroxide and telomere length in OSA patients.** A significant inverse correlation was observed between the concentration of hydrogen peroxide and the telomere length of 43 OSA patients (R^2^=0.490) (A) and mean telomere length in healthy volunteers (N = 34) and OSA patients (N = 43) (B). Graphs show mean values. White dot: telomere length of healthy volunteers; black dot: telomere length of OSA patients (^*^p < .05 comparing healthy volunteers and OSA patients).

### Shorter telomere length was observed in circulating leukocytes in the peripheral blood of OSA patients

**Figure 3.**
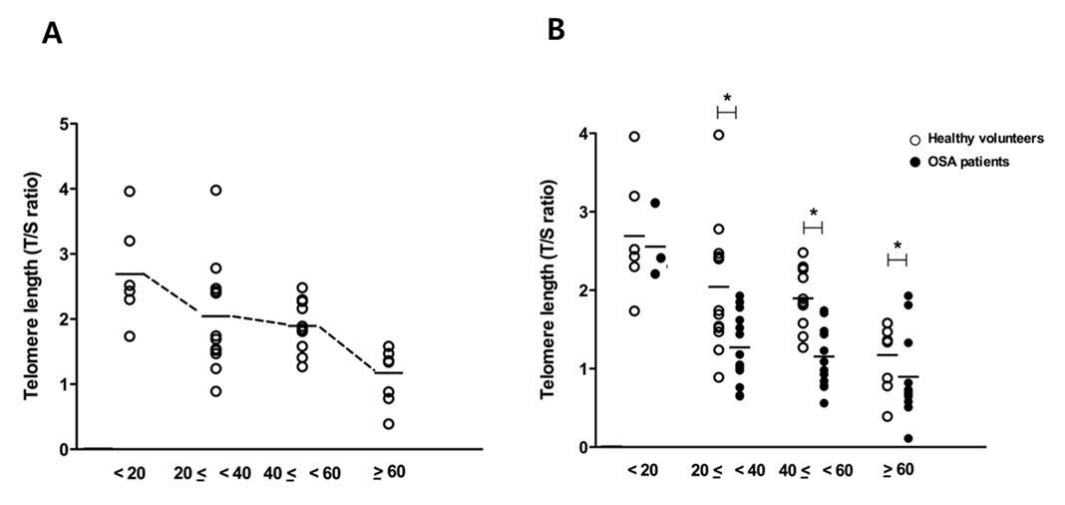
**Change in telomere length by age.** Mean values of telomere length diminished with age in healthy volunteers (N=34), and the lowest values were observed in people older than 60 years (five were younger than 20 years old, twelve were 20-40 years, ten were 40-60 years, and seven were older than 60 years) (A). Mean values were significantly shorter in OSA patients (N=43, three who were younger than 20 years, sixteen were 20-40 years, thirteen were 40-60 years, and eleven were older than 60 years) than in healthy volunteers of the same age (B). Graphs show mean values. White dot: telomere length of healthy volunteers; black dot: telomere length of OSA patients. ^*^p <.05 comparing healthy volunteers and OSA patients.

We compared the change in telomere length with the hydrogen peroxide concentration of the peripheral blood in OSA patients to investigate potential associations between the two factors. Interestingly, we found a predictable inverse correlation between hydrogen peroxide concentration in the peripheral blood and telomere length of circulating leukocytes (R^2^=0.490, Fig. 2A). As a next step, we measured telomere length in circulating leukocytes from patients with OSA and healthy volunteers and the results showed that mean telomere length was significantly shorter in the DNA from whole blood samples of OSA patients compared to healthy volunteers (1.87 ± 0.74 vs. 1.14 ± 0.60, *p* < 0.05) (Fig. 2B). Then, we analyzed the change in telomere length in an age- and BMI-dependent manner, because telomere length is also influenced by age and body mass index (BMI). Telomere length of circulating leukocyte in whole blood DNA of healthy volunteers shortened in accordance with advancing subject’s age. Mean telomere length was 2.69 ± 0.7 in circulating leukocytes of healthy volunteers younger than 20 years (N=6), 2.04 ± 0.8 in healthy volunteers aged 20-40 years (N=12), 1.89 ± 0.4 in healthy volunteers aged 40-60 years (N=10), and 1.17 ± 0.4 in healthy volunteers older than 60 years (N=6) (Fig. 3A).

Mean telomere length from whole blood DNA was compared between participants with and without OSA according to age. The mean telomere length from whole blood DNA of OSA patients was considerably shorter in those over 20 years age compared to that of healthy volunteers (2.04 ± 0.8 in healthy vs. 1.27 ± 0.4 in OSA for 20-40 years (OSA patients, N=16); 1.89 ± 0.4 vs. 1.15 ± 0.3 for 40-60 years (OSA patients, N=13); 1.17 ± 0.4 vs. 0.89 ± 0.5 for over 60 years (OSA patients, N=11); Fig. 3B). The results suggested that significantly shorter telomere lengths were observed in the peripheral blood of OSA patients and these changes progressed more rapidly in an age-dependent manner, compared to healthy volunteers.

**Figure 4.**
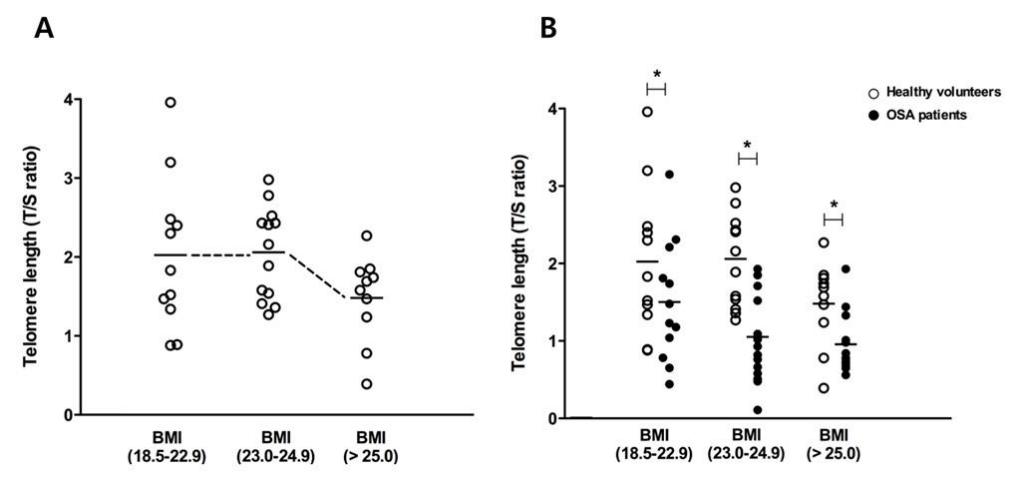
**Change in telomere length by body mass index (BMI).** Mean telomere length diminished with higher BMI, and the lowest value was observed for BMI > 25 cm^2^/kg in healthy volunteers (eleven were less than 22.9 kg/cm^2^, thirteen were 23.0 to 24.9 kg/cm^2^, and ten were greater than 25.0 kg/cm^2^) (A). Mean values were significantly shorter in OSA patients (twelve were less than 22.9 kg/cm^2^, seventeen were 23.0 to 24.9 kg/cm^2^, and fourteen were greater than 25.0 kg/cm^2^) than in healthy volunteers in the same BMI range (B). Graphs show mean values. White dot: telomere length of healthy volunteers; black dot: telomere length of OSA patients. ^*^p <.05 comparing healthy volunteers and OSA patients.

The mean telomere length was 2.02 ± 0.9 in those with a BMI less than 22.9 kg/cm^2^ (N=11), 2.05 ± 0.8 in people with a BMI 23.0 to 24.9 kg/cm^2^ (N=13), and 1.48 ± 0.5 in people with a BMI greater than 25.0 kg/cm^2^ (N=10) (Fig. 4A). Thus, we found that telomere length from the whole blood DNA of healthy volunteers also shortened if subjects had greater BMI.

Mean telomere lengths from the whole blood DNA of OSA patients were compared with those of healthy volunteers according to BMI category. The mean telomere length from the whole blood DNA of OSA patients was considerably shorter in all BMI categories compared to healthy volunteers (2.02 ± 0.9 healthy vs. 1.50 ± 0.8 for OSA below BMI 22.9 kg/cm^2^ (OSA patients, N=12); 2.05 ± 0.8 vs. 1.05 ± 0.4 for BMI from 23.0 to 24.9 kg/cm^2^ (OSA patients, N=17); 1.48 ± 0.5 vs. 0.95 ± 0.4 for BMI over 25.0 kg/cm^2^ (OSA patients, N=14); Fig. 4B). These results indicated that telomere length also decreased more acutely in a BMI-dependent manner for OSA patients compared to healthy volunteers.

### Telomere length in OSA patients correlated with OSA severity and oxygen desaturation

Based on the RDI, OSA patients were classified into three groups by OSA severity. Among 43 OSA patients, eleven were classified with mild, sixteen with moderate, and sixteen with severe OSA.

**Figure 5.**
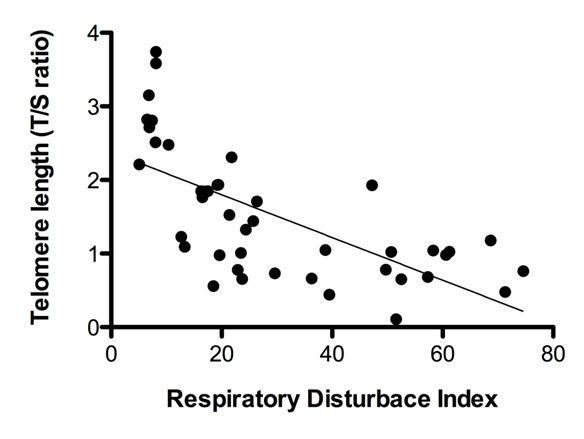
**Comparison of telomere length in the circulating leukocytes in the peripheral blood of OSA patients (N = 43) depends on OSA severity.** A significant inverse correlation was observed between telomere length and the RDI of 43 OSA patients (R^2^=0.446).

**Figure 6.**
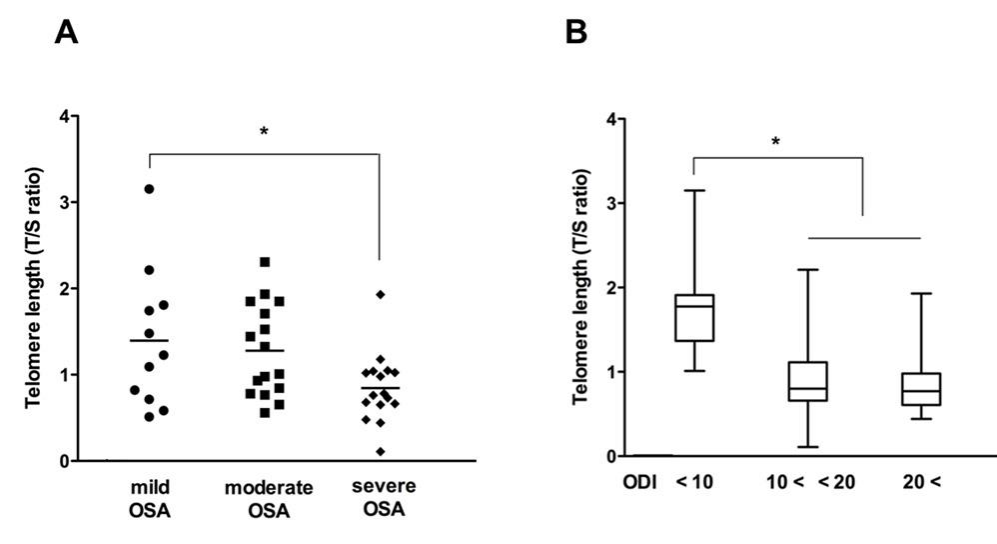
**Comparison of telomere length in the circulating leukocytes in the peripheral blood of OSA patients (N = 43) depends on OSA severity.** The telomere length of circulating leukocytes was significantly shorter in patients with severe OSA than patients with mild OSA (11 mild OSA (circle), 16 moderate OSA (rectangle), and 16 severe OSA (diamond) patients). Graphs show mean values, and ^*^p < .05 compared to patients with mild OSA (A). Telomere length of circulating leukocytes in OSA patients with >20 ODI was significantly shorter than in OSA patients with <10 ODI (B). Graphs show mean value + standard deviation (SD). ^*^p < .05 compared to OSA patients with <10 ODI.

A significant inverse correlation was observed between telomere length and the RDI of forty-three OSA patients (R^2^=0.446, *p* < 0.05, Fig. 5). The mean telomere length in samples from the eleven patients with mild OSA was 1.40 ± 0.8; this value was significantly decreased in patients with severe OSA (1.07 ± 0.5, *p* < 0.05, Fig. 6A). OSA patients were also classified by oxygen desaturation index (ODI) dependence, and mean values of telomere length were compared according to ODI. Twelve OSA patients had an ODI lower than 10; eighteen had an ODI from 10 to 20; and thirteen had an ODI greater than 20. The mean telomere length was shorter in patients with an ODI greater than 20 compared to patients with an ODI less than 10 (0.89 ± 0.5 greater than 20 vs. 1.75 ± 0.6 less than 10; Fig. 6B). These results revealed that telomere shortening is related to OSA severity as shorter telomere lengths were seen in OSA patients with a greater number of apnea events and lower oxygen desaturation indexes.

## DISCUSSION

This study shows that OSA patients present with increased levels of systemic oxidative stress compared to healthy volunteers and distinctive telomere length shortening of circulating leukocytes is closely related to the burden of oxidative stress of the peripheral blood from OSA patients.

Studies done several years ago found that approximately 40% patients with severe OSA died during a follow-up period of 8 years with the main pathology behind OSA being airway collapse, with many contributing anatomical factors [12-14]. Airway collapse can provoke increased intermittent hypoxia and oxidative stress in the blood of people with OSA [2,4,9]. The reperfusion/re-oxygenation that follows the hypoxic period during an episode of apnea or hypopnea activates a variety of cells including endothelial cells, leukocytes, and lymphocytes, which propagate inflammatory processes resulting in free radical production [15,16]. We presume that OSA patients are exposed to repetitive apneic episodes that may cause systemic oxidative stress in the blood without proper treatment. In addition, OSA is characterized by long-term sleep deprivation, which activates lipid peroxidation and inhibits antioxidant defense systems [16]. Therefore, we estimated that intermittent hypoxia during sleep is the major trigger factor for free oxygen radical production in sleep apnea and excessive production of free radicals may be a pathophysiological process which contributes to induce systemic oxidative stress in OSA patients [17,18]. Indeed, our data showed that the concentration of hydrogen peroxide which might be a main free radical was considerably higher in the peripheral blood of OSA patients than that of healthy volunteers, providing evidence that the burden of oxidative stress might be significantly elevated in OSA patients due to apnea events during sleep. Prior studies proposed that systemic oxidative stress is interrelated to cardiovascular diseases and these are the most common complications which can be arised from untreated OSA [19-21]. Sleep studies are effective to diagnose OSA itself and estimate the severity of OSA but cannot predict the burden of oxidative stress in OSA patients. Therefore, we thought that the critical biomarkers need to be developed to assess the systemic oxidative stress in the peripheral blood of OSA patients. The clinical researches assessing biomarkers may promote a better estimate of the burden of oxidative stress and lead to improved therapeutic strategies for managing the cardiovascular complications of OSA patients.

Telomere length reflects the degree of systemic inflammation, and telomere attrition may be relevant to age-related or chronic inflammatory diseases [10-12]. Cumulative lifetime inflammatory burdens may be important for inducing abnormal cellular aging and unbalanced oxidative stress can cause excessive cellular aging, resulting in a higher prevalence of chronic inflammatory diseases [22, 23].

Some studies suggested the contribution of oxidative stress to shortening leukocyte telomere length, as telomere length has been negatively correlated with plasma oxidative stress and cellular DNA damage [3,11]. The present findings extended those studies and showed that telomere length measured using the genomic DNA of circulating leukocytes in the peripheral blood of OSA patients was significantly shorter than that of healthy volunteers. In addition, shortening of telomere length was more pronounced in OSA patients with higher RDI and oxygen desaturation index.

We measured the hydrogen peroxide concentration in the peripheral blood of OSA patients and observed that it was inversely related to the telomere length of circulating leukocytes. Our data indicated that the degree of oxygen desaturation is relatively related to the amount of intermittent hypoxia and may be representative of oxidative stress levels in the peripheral blood of OSA patients.

Prospective studies have shown that untreated OSA is associated with increased mortality, and airway collapse must be controlled to avoid the fatal complications of OSA [24, 25]. We thought that oxidative stress caused DNA impairment or epigenetic modifications of nuclear DNA in circulating blood cells beyond a critical value and an imbalance of oxygen consumption in the blood of OSA patients might promote harmful stimulation and cellular damage to whole blood cells, leading to excessive telomere shortening. The higher prevalence of cardiovascular complications in OSA patients may account for increased mortality with a few clinical biomarkers that may represent the burden of cardiovascular risk [24-26]. It has been reported that the release of tumor necrosis factor-alpha and interleukin-8 production from peripheral blood in patients suspected OSA was not different compared to non-OSA subjects and in patients showing similar prevalence of major cardiovascular risk factors and cardio-metabolic therapies, differing for the presence or absence of OSA, cytokine productions from peripheral blood were similar [27]. Therefore, it would be hard to find effective clinical markers to predict the risk of cardiovascular complications. In the current study, we concentrated on the degree of oxidative stress of OSA subjects and oxidative stress-related clinical markers to represent the cardiovascular burden. We showed that oxidative stress influences telomere length shortening in OSA patients and found a correlation between telomere length and apnea events or oxygen desaturation in OSA patients such that telomere length shortening was closely related to OSA severity. Accordingly, we believe that the precise measurement of biomarkers for cellular aging, such as telomere length may harbor potential utility to predict the OSA progression or severity and changes in telomere length of circulating leukocytes from OSA patients may be used to prognosticate adverse outcomes due to cardiovascular complications. Moreover, telomere length may also provide predictive insight into the significance of oxidative stress in peripheral blood and cardiovascular risk in OSA subjects who are not treated appropriately.

Age and BMI are common factors that influence changes in cellular aging [2]. This study analyzed telomere length in an age- and BMI-dependent manner for both healthy volunteers and OSA patients. The data demonstrated that telomere length was considerably shorter in OSA patients than in age- and BMI-matched groups of healthy volunteers. We propose that elderly and obese OSA patients require more efficient and prompt treatment of airway collapse in order to prevent the aggravation of cardiovascular complications. Telomere length in the peripheral blood should be assessed as a possible predictive marker of the progression of cardiovascular diseases in all OSA patients.

In summary, this current study demonstrates that telomere length was shorter in the circulating leukocytes of patients with OSA than in healthy volunteers and appeared to be dependent on severity of OSA and the degree of systemic oxidative stress. These findings also suggest that telomere length of circulating leukocytes may be a reliable biomarker to predict the life-time burden of oxidative stress from OSA.
